# Looking to the Future of Viral Vectors in Ocular Gene Therapy: Clinical Review

**DOI:** 10.3390/biomedicines13020365

**Published:** 2025-02-05

**Authors:** Chulpan B. Kharisova, Kristina V. Kitaeva, Valeriya V. Solovyeva, Albert A. Sufianov, Galina Z. Sufianova, Rustem F. Akhmetshin, Sofia N. Bulgar, Albert A. Rizvanov

**Affiliations:** 1 Institute of Fundamental Medicine and Biology, Kazan Federal University, 420008 Kazan, Russia; harisovachulpan@gmail.com (C.B.K.); krvkitaeva@kpfu.ru (K.V.K.); vavsoloveva@kpfu.ru (V.V.S.); 2 Department of Neurosurgery, Sechenov First Moscow State Medical University (Sechenov University), Ministry of Health of the Russian Federation, 119991 Moscow, Russia; sufianov_a_a@staff.sechenov.ru; 3 Federal State-Financed Institution “Federal Centre of Neurosurgery”, Ministry of Health of the Russian Federation, 625032 Tyumen, Russia; 4 Department of Pharmacology, Tyumen State Medical University, 625023 Tyumen, Russia; sufarm@mail.ru; 5 The Department of Ophthalmology, Kazan State Medical University, 420012 Kazan, Russia; rustemfa@mail.ru; 6 Kazan State Medical Academy—Branch Campus of the Federal State Budgetary Educational Institution of Further Professional Education, Russian Medical Academy of Continuous Professional Education, Ministry of Healthcare of the Russian Federation, 420012 Kazan, Russia; sofia_spring@mail.ru; 7 Republican Clinical Ophthalmological Hospital of the Ministry of Health of the Republic of Tatarstan, 420012 Kazan, Russia; 8 Division of Medical and Biological Sciences, Tatarstan Academy of Sciences, 420111 Kazan, Russia

**Keywords:** ocular diseases, gene therapy, viral vectors, retinal degeneration, optic nerve disorder, cornea diseases

## Abstract

Eye diseases can significantly affect the quality of life of patients due to decreased visual acuity. Although modern ophthalmological diagnostic methods exist, some diseases of the visual system are asymptomatic in the early stages. Most patients seek advice from an ophthalmologist as a result of rapidly progressive manifestation of symptoms. A number of inherited and acquired eye diseases have only supportive treatment without eliminating the etiologic factor. A promising solution to this problem may be gene therapy, which has proven efficacy and safety shown in a number of clinical studies. By directly altering or replacing defective genes, this therapeutic approach will stop as well as reverse the progression of eye diseases. This review examines the concept of gene therapy and its application in the field of ocular pathologies, emphasizing the most recent scientific advances and their potential impacts on visual function status.

## 1. Introduction

According to the World Health Organization (WHO), the global prevalence of visual impairment in 2023 exceeded two billion individuals, with approximately half of these cases deemed to be preventable [1]. The predominant etiologies of visual impairment and blindness encompass refractive anomalies, cataracts, diabetic retinopathy, glaucoma, and age-related macular degeneration (AMD) [2].

Gene therapy has become a rapidly expanding field, with significant advances in recent years. There are four main strategies in gene therapy that can prevent the progression of a wide range of diseases of the visual system. These are as follows: the delivery of a normally functioning gene (wild-type gene), the suppression of mutated gene expression, the addition of genes to increase expression and affect cell function, and gene editing [3]. Consequently, gene therapy has emerged as a promising therapeutic modality for addressing fundamental genetic and molecular pathologies, paving the way for novel therapeutic interventions for various ocular diseases.

## 2. Gene Therapy Approaches for Diseases of the Visual System

Gene therapy for ophthalmic diseases constitutes a treatment modality involving the transfer of genetic material into specific cells via vectors. The primary vectors employed in the context of gene therapy for ocular diseases encompass adenovirus (Ad), adeno-associated virus (AAV), and lentivirus (LV) [4]. Non-viral vectors are a diverse group of chemical and physical methods of delivering genetic material into cells. For instance, the delivery of targeted genes into the anterior chamber of the eye can be accomplished by employing non-viral vectors, including naked DNA, nanoparticles, and nanopolymers (metal, magnetic, micellar, and liposomal nanoparticles, cationic nanoparticles, nanopolymers, and dendrimers), microinjection, electroporation, sonoporation, iontophoresis, gene gun, laser, chemical, antisense oligonucleotides, and siRNA [5]. In this section, the primary properties, advantages, and disadvantages of individual viral and non-viral vectors will be discussed, as well as FDA-registered gene therapeutic drugs based on them.

### 2.1. Viral Vectors

The potential of gene therapy is significant due to the capacity for directed delivery of functional gene copies to target organs and tissues. This can be achieved using a modern and versatile tool, namely, a viral vector.

#### 2.1.1. Adenoviruses

The Ad vector has several advantages, and understanding the tropism of different Ad serotypes to different cells in the eye will greatly accelerate the development of adenovirus-mediated gene therapy for the eye [6]. Despite several advantages, Ad vector gene therapy studies have demonstrated the occurrence of a strong immune response leading to systemic inflammation, which reduces the efficiency of vector transduction and shortens the duration of transgene expression [7].

The efficacy of Ad vectors encoding the pigment epithelium-derived factor (PEDF) gene has been demonstrated in preclinical models of age-related macular degeneration and diabetic retinopathy [8]. In addition, Ad vectors are currently employed in anti-tumor therapy. A phase I clinical trial is currently underway to assess the safety and efficacy of the oncolytic adenovirus VCN-01 in patients with refractory retinoblastoma, a retinal tumor [NCT03284268].

#### 2.1.2. Adeno-Associated Viruses

The AAV is the most frequently utilized vector in the domain of ocular gene therapy research, owing to its established safety profile and the capacity for prolonged and stable expression. AAV-based gene therapy clinical studies for ocular diseases, utilizing AAV2 and AAV8, have yielded encouraging results [9,10,11,12,13].

It is noteworthy that Luxturna^®^ (voretigene neparvovec-rzyl), developed by the US-based company Spark Therapeutics, Inc., has caused a significant breakthrough in the field of gene therapy for diseases of the visual system. It is the first AAV2-based gene therapy to be FDA-approved, and it accomplishes visual cycle restoration by delivering a functional *RPE65* gene, which codes for retinoid isomerohydrolase, to retinal pigment epithelial cells. This treatment is intended for individuals diagnosed with Leber congenital amaurosis (LCA) RPE65 [14].

AAVs have been demonstrated to transduce not only actively dividing cells but also those in a quiescent state, including retinal cells. The latent state of viral particles within the host cell DNA is maintained by their integration into specific chromosomal loci (AAV integration sites). This integration is subject to disruption by the presence of a helper virus, which can stimulate the replication of the viral particles [15,16]. An alternative way to deliver large genes via AAV is to create double vectors [17]. The efficacy of co-transduction by two or more AAV vectors has been demonstrated by subretinal injection into retinal cells [18], mouse models of Stargardt disease, and Usher syndrome type IF [19,20].

Furthermore, the capsid, the genome, and the transgene product are the primary components of AAV vectors that have the potential to elicit an immune response [21]. Like Ad vectors, AAV vectors can activate a humoral immune response triggered by natural infections or by Ad or AAV gene transfer [22]. Therefore, the efficiency of gene transfer can be considerably impacted by neutralizing antibodies (Abs) directed against a particular serotype [13]. To date, several strategies have been developed to mitigate the immunogenicity of AAVs. These strategies include the following: reducing the vector dose, administering corticosteroids, implementing strict screening criteria, and excluding AAVs from clinical trials [23].

Following the approval of the inaugural AAV-based pharmaceuticals, a plethora of clinical trials employing this vector have been registered, surpassing 200 in number. Thus, 2 phase I/II clinical trials [NCT02599922] to evaluate the safety and efficacy of gene therapy for achromatopsia in patients with a mutation in the cyclic nucleotide gated channel gene (*CNG*)-B3 have been registered, one of which has been completed [NCT03001310]. AAV2 was used in these studies. Phase III and II clinical trials of choroideremia gene therapy based on AAV2 encoding the *REP1* gene [NCT03496012, NCT02671539, and NCT02407678], known in humans as CHM [NCT02341807], have also been completed. Table 1 summarizes all current clinical trials for visual diseases based on AAV.

#### 2.1.3. Lentiviruses

LV vectors are integrating vectors that provide long-term transgene expression. These vectors possess a relatively modest target gene insertion volume, with a maximum of 8 bp [24]. Nonetheless, the implementation of two distinct vectors carrying interdependent transgenes may not represent an optimal solution, as successful transduction of multiple viral vectors into a singular cell is not efficient [3]. LVs have been shown to have the ability to express multiple genes from a single vector [25]. The primary drawbacks associated with the utilization of LV vectors in gene therapy include the occurrence of recombination and insertional mutagenesis. LVs exhibit a high recombination frequency and mutate rapidly, which can lead to superinfection [26,27]. The LV RNA genome can also activate the innate immune response [28]. To date, it is possible to reduce such risks by distributing the core genes of the original viruses into separate plasmids [29]. The second major risk in the use of retroviral vectors is insertional mutagenesis, which triggers oncogenesis [30]. However, LV is a more secure option in this regard as it is integrated into the genome due to the presence of transcriptionally active sites, as opposed to being inserted randomly [31].

RetinoStat^®^ (OXB-201), the precursor to OXB-203, has now been demonstrated to be an LV vector based on equine infectious anemia virus expressing endostatin (ES) and angiostatin (ANG). This gene product is intended to treat neovascular AMD by inhibiting the development of choroidal neovascularization [32]. In a phase I study involving 21 patients [NCT01301443] and a follow-up safety assessment study [NCT01678872], RetinoStat^®^ demonstrated stable and long-term protein expression in the aqueous humor of patients up to 6 years after a single subretinal injection.

Beyond the scope of gene delivery and gene expression restoration, a range of other gene editing techniques exists. However, ZFN and TALEN have not found mass application in medicine due to the complexity and labor-intensive assembly and production of these systems [33,34]. CRISPR technology directs Cas proteins to a specific location in the genome by altering the base sequence of a small segment of guide RNA, thereby increasing the efficiency of gene editing and expanding the potential applications [35]. A common challenge is that each CRISPR-Cas-related therapeutic must deliver large amounts of genome-editing enzyme into cells, and sometimes simultaneous delivery of multiple macromolecules is required. Studies have demonstrated that CRISPR-Cas tools may be more susceptible to off-target effects than other traditional gene editing methods [36]. The delivery of CRISPR systems in vivo can induce immune responses. A major challenge is that humans may be pre-exposed to the same Cas nuclease effector antigens, and/or delivery vectors are required to carry the effectors for targeted treatment. To date, only three clinical trials of therapy using the CRISPR/Cas9 genome editing therapy have been reported for retinitis pigmentosa (RP) [NCT05805007], Leber’s congenital amaurosis type 10 [NCT03872479], and refractory viral keratitis [NCT04560790].

#### 2.1.4. Non-Viral Vectors

Non-viral vectors involve gene delivery by both chemical carriers and physical methods [37,38]. These cells possess a distinct advantage due to their ease of manipulation, allowing for the modification of their properties to suit the specific needs of the target cell and the intended purpose. Additionally, the production cost associated with their use is relatively low.

Physical methods of transferring genetic material are simple and rely on the use of physical force to affect the cell membrane, facilitating intracellular delivery of genetic material. These methods include DNA delivery by micro-needle injection [39], ballistic DNA injection [40], electroporation [41], sonoporation [42], photoporation [43], magnetofection [44], and hydroporation [45]. In comparison with viral vectors, non-viral vectors exhibit reduced toxicity, immunogenicity, and mutagenesis. However, despite these advantages, non-viral vectors are employed in gene transfer with low efficiency, specificity, duration of gene expression, and safety [46].

## 3. Methods of Addressable Vector Delivery

**Figure 1:**
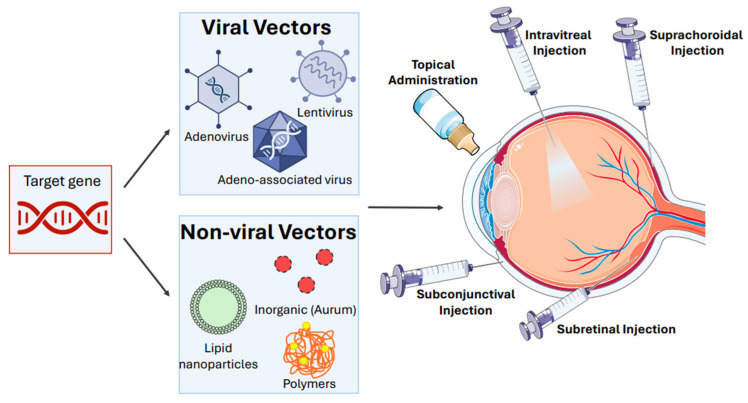
Types of targeted gene delivery for gene therapy of ocular diseases.

The eye has many barriers, such as the corneal and conjunctival epithelial, blood-aqueous, and blood-retinal barriers [47]. There are four main invasive methods of injecting vector delivery: intravitreal, subretinal, suprachoroidal, and subconjunctival methods [47,48]. As illustrated in Figure 1, there are two broad categories of gene delivery methods: viral and non-viral. The gene therapy products can be administered via various routes.

### 3.1. Subretinal Method of Administration

Subretinal injections are the most studied and the only method of vector administration for transduction cells of retinal pigment epithelium and photoreceptor cells; other delivery methods are not available to target these cell types [38]. The FDA-approved drug Luxturna^®^ for the treatment of hereditary retinal dystrophy is administered by the described subretinal injection method [49].

### 3.2. Intravitreal Injection Method

The intravitreal method of administration has been demonstrated to be the most applicable method of drug delivery in clinical practice. This approach is characterized by its minimal invasiveness, reduced incidence of complications, and the capacity to ensure a more uniform distribution of viral vectors over the retina. Another advantage of gene delivery into the vitreous body is its theoretical capacity to transduce the entire retinal surface, as opposed to the subretinal method of administration [4]. Evidence has demonstrated the efficacy of this method of administration in the treatment of optic nerve and lens pathologies [38]. Using intravitreal injection, IZERVAY™ is an FDA-approved drug for the treatment of geographic atrophy [50].

### 3.3. Suprachoroidal Method of Administration

The primary benefit of suprachoroidal delivery is its enhanced targeting and bioavailability across a more extensive area of the retina and vasculature in comparison with intravitreal injections. In this novel approach, a suspension of vectors is injected into the space between the sclera and choroid [51]. However, the efficacy of retinal cell transduction after suprachoriodal injection may be hampered by rapid choriocapillary clearance [51]. ABBV-RGX-314, a gene-therapeutic drug, is being developed by RegenxBio Inc., which is administered suprachoroidally. A phase II clinical trial [NCT05407636] demonstrated safety and evidence of a dose-dependent response in patients with wet age-related macular degeneration.

### 3.4. Periocular Method of Administration

The administration of periocular injections can be achieved through various methods, including peribulbar, retrobulbar, posterior juxtascleral, sub-tenon, and subconjunctival injection [47]. Subconjunctival injections of AAV vectors have been demonstrated to transduce the eyelid, conjunctiva, cornea, optic nerve, and periocular tissues, including muscle [52]. Nevertheless, the injected pharmaceutical agent has the potential to enter the systemic bloodstream, thereby limiting its ocular bioavailability [53].

### 3.5. Physical Methods of Delivery

Physical methods of delivering viral vectors to tissues also include electroporation, which uses short, high-voltage electrical pulses to create pores in the lipid bilayer of the cell membrane for the passage of genetic material [41]. In conjunction with alternative injection methodologies, specific cells can be targeted, including photoreceptors, retinal pigment epithelium, and retinal ganglion cells (RGCs). For instance, by integrating subretinal injection of genetic material with electroporation, photoreceptors or the retinal pigment epithelium can be targeted by adjusting the direction of the electrical pulses [54]. However, in the posterior region of the eye, such as the retina, invasive electrode placement surgery is necessary because proximity to the target cells is required to generate a localized and effective electric field [38]. This creates the problem of transferring electroporation from preclinical studies in laboratory animals to clinical practice in humans

A phase I/II clinical trial, pEYS606 [NCT03308045], was completed in 2022 to evaluate the safety and tolerability of electroporation into the ciliary muscle of a plasmid encoding chimeric TNF-α receptor (hTNFR-Is) for the therapy of non-infectious uveitis.

## 4. Physiological Barriers of the Eye for Drug Delivery

The biological barriers of the eye can be classified into three categories: precorneal, corneal, and blood–ocular barriers.

The main component of the precorneal barrier is the nasolacrimal system. The consequence of this phenomenon is twofold: first, it results in the removal of the liquid dosage form of the injected preparation, and second, it reduces the exposure time of the cornea. In addition, increased lacrimal flow may lead to faster excretion of precorneal fluid and decrease therapeutic efficacy. The osmolarity and pH of the pharmaceutical agent under consideration are two important parameters that have the potential to induce an increase in tear fluid formation [55]. Furthermore, tears contain a high concentration of proteins, which may interact with the drug, thereby altering its bioavailability [55,56].

The cornea is composed of multiple layers and is a multifaceted barrier. It contains lipophilic structures, such as the epithelium, which consists of six layers, and the endothelium. In addition, it contains hydrophilic structures, including the stroma. Furthermore, the corneal barrier is composed of intercellular tight junctions (*zonula occludens*), which envelop the surface epithelial cells. These junctions act as a selective filter for small molecules, thereby completely preventing the diffusion of macromolecules via paracellular transport [57]. Consequently, lipophilicity, solubility, molecular size, charge, and degree of ionization significantly impact the penetration of the active substance into the cornea, determining its rate and pathway.

The blood–ocular barriers constitute a biological filtration system that is situated between the blood vessels and the interior of the eye. These proteins represent a significant impediment to the systemic and local delivery of pharmaceutical agents to the anterior and posterior chambers of the eye. The blood–aqueous barrier is formed by the following elements: non-pigmented ciliary epithelial cells, endothelium of iris blood vessels, and endothelium of the inner wall of the Schlemm’s canal [58]. This barrier fulfills a key role in the regulation of aqueous moisture homeostasis by selectively restricting the passage of plasma proteins into the aqueous environment [59]. The permeability of the blood–aqueous barrier depends on the diffusion pressure associated with transport activity [58]. The blood–retinal barrier consists of both an inner and an outer barrier [60]. Disruption of the outer barrier leads to an increase in vascular capillary permeability, which causes macular edema seen in various retinopathies [61]. Disruption of the inner blood–retinal barrier can be caused by acute stretching of the vessel walls, ischemia, chemical exposures, endothelial cell defects, or transport system deficiencies [61]. The retinal tissue itself lacks a barrier in its stroma, thereby enabling fluid to diffuse from one part to neighboring areas.

## 5. Challenge of Preclinical and Clinical Trials of Gene Therapy

To date, 15,276 clinical trials of various approaches in the therapy of eye diseases have been registered, of which 143 are evaluating the safety and efficacy of gene therapy drugs. In this section, we will consider the most current characteristics for retinal, cornea, and optic nerve pathologies. We will also examine narrowly focused clinical trials of gene therapy for pathologies of the visual system. Finally, we will discuss etiological and pathogenetic factors of these diseases. Table 1 demonstrates the clinical trials registered worldwide.

**Figure 2:**
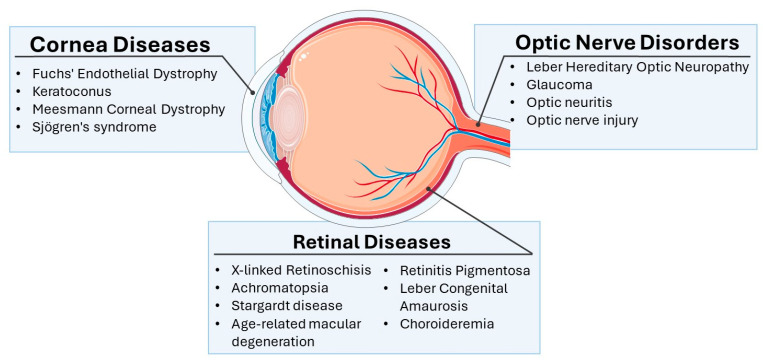
The most relevant diseases for gene therapy depend on the section of the eye.

This section will provide a comprehensive review of the major categories of ocular diseases that have become targets for gene therapy development. As illustrated in Figure 2, ocular diseases manifest in various regions of the anatomical structures of the eye.

### 5.1. Optic Nerve Lesions

The presence of optic nerve abnormalities is an indirect indication of damage to the brain or the maxillofacial region. Damage may be characterized by the loss of RGCs and axons, which can result in abnormal pupillary light reflexes, visual field defects, and vision loss [62].

**Figure 3:**
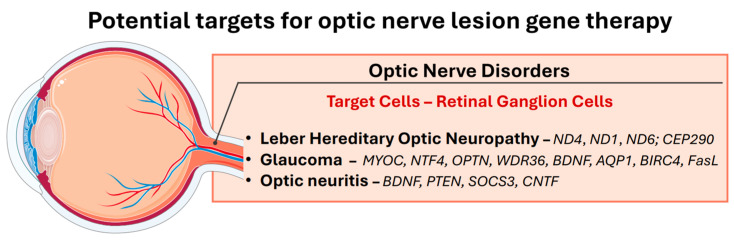
A gene therapy strategy for optic nerve pathologies.

A variety of optic nerve pathologies have the potential to be treated with gene therapy: Leber’s hereditary optic neuropathy, glaucoma, and optic neuritis [63]. There are two main approaches by which gene therapy can be feasible in the treatment of these diseases. It can be used to correct a specific gene defect in conditions where the defect is well understood. Second, gene therapy can be used to alter gene expression in such a way as to slow the course of the disease or provide some form of prevention against possible complications of the disease. The primary objectives of developing gene therapy for optic nerve pathologies are outlined in Figure 3.

#### 5.1.1. Leber’s Hereditary Optic Neuropathy

Leber’s hereditary optic neuropathy (LHON) is the most common inherited mitochondrial disease, characterized by bilateral vision loss in early adulthood. Its incidence is 1:30,000–50,000 people. This pathology affects RGCs, the axons of which form the optic nerve, leading to its atrophy and profound vision loss [64]. LHON results from a mutation in mitochondrial DNA in the region encoding nicotinamide adenine dinucleotides ND4, ND1, and ND6, a subunit of respiratory complex 1 [65]. Currently, patients with this disease receive only maintenance treatment based on antioxidant therapy, taking idebenone. This therapeutic approach has demonstrated safety and limited overall efficacy, depending on the stage and debut of the disease [66].

In preclinical trials, a major challenge in understanding the mechanisms of different forms of LHON is the lack of access to human tissues to study these cells in vitro and the limitations of existing in vivo animal models. Blood cells, fibroblasts, cybrids, and RGC models derived from induced pluripotent stem cells (iPSCs) [67] are used as a model for studies of mitochondrial DNA content. Certainly, iPSC-derived models have distinct advantages [68]. RGCs are a cell type that includes diseased tissue, but iPSC derivation and differentiation, as well as multiple mutations, introduce limitations in the use of this model in preclinical trials of LHON therapy. A mouse line with a respiratory complex I deficiency model was developed for drug testing by Zhang et al. [69]. This model was obtained by intravitreal injection of a respiratory complex I inhibitor (rotenone) into the eyes of mice. The effect corresponded to the degeneration of RGCs.

The results of preclinical gene therapy studies formed the basis for the launch of three large clinical trials of LHON in the US [NCT02161380], Europe [NCT02064569], and China [NCT01267422]. Each study was designed to restore wild-type human *ND4* expression in patients carrying the most common variant of LHON.

Gene therapy is a rational approach for the treatment of LHON. However, the development of gene therapy for mitochondrial diseases has been hindered by the complexity of delivering genes to mitochondria and the absence of adequate preclinical models for clinical trials. In 2015, GS010 (lenadogene nolparvovec, GenSight) received FDA approval for participation in phase III studies, having completed clinical testing in three pivotal trials, NCT02652767, NCT02652780, and NCT03293524. Despite visual improvements beyond expected outcomes, overall efficacy was limited and complicated by unexpected similar improvements in the healthy eye. The contralateral effect observed in this study may be attributed to the natural progression of the disease, the mechanical transfer of viral vector DNA from one eye to the other, or a learning effect resulting from the clinical trial design and visual acuity determination methods employed [70].

In addition, one of the methods of gene therapy for LCA in a mutation in the *CEP290* gene is intravitreal injection of AON-sepofarsen, which leads to persistent suppression of pathological RNA transcripts by skipping exons [71,72]. Phase I/II clinical trials have demonstrated safety, improved visual acuity, and increased retinal sensitivity to light, supporting the continued development of AON. However, a double-blind, randomized controlled phase II/III study evaluating the efficacy, safety, tolerability, and systemic effects of intravitreal injections of AON (QR-110) in CEP290-LCA did not meet the clinical endpoint [NCT03913143].

Despite promising progress in gene therapy for LHON, this gene therapy approach is not yet approved; thus, further efficacy studies in restoring ND4 expression and function are needed.

#### 5.1.2. Glaucoma

Glaucoma is a complex polygenetic disease, an optical neuropathy resulting from optic nerve damage caused by elevated intraocular pressure (IOP). Since the main therapeutic strategies—pharmacologic, surgical, and laser methods aimed at IOP elevation—do not restore lost vision, it is expected that new modern therapies will be developed.

This disease is characterized by degeneration of RGCs and loss of their axons in the optic nerve, resulting in irreversible blindness [73]. Neurodegeneration due to glaucoma is often preceded by decreased ocular perfusion due to decreased blood flow, hypoxia, and oxidative stress, which are inducers of autophagy [74,75]. It is imperative to acknowledge that not all individuals diagnosed with glaucoma exhibit IOP; normal pressure glaucoma is also distinguished. Thus, ischemia and histopathologic glaucomatous abnormalities can occur in both elevated IOP and normal IOP conditions [76]. Common to these conditions is the degeneration of RGCs.

The classification of glaucoma is determined by its etiology and the time of disease onset. Glaucoma is thus categorized into three distinct classifications: primary, secondary, and congenital. The most common type worldwide is primary open-angle glaucoma (POAG) [77]. A whole-genome association study revealed that four pathogenic genes, *MYOC*, *NTF4*, *OPTN*, and *WDR36*, are associated with closed-angle glaucoma (CAG) [78]. In a study by Han et al., large-scale genome-wide association analyses identified 263 loci for POAG in a group of European ancestry [79].

Of course, before starting the development of a gene product for glaucoma therapy, it is necessary to select both in vitro and in vivo models of the disease. Rodents are frequently utilized as models for pre-clinical trials for glaucoma therapy. A limitation of these results is the current lack of understanding of the trabecular network of the human and mouse eye, which hinders the ability to adequately represent these results to humans. It has been confirmed that human and mouse cells of the ocular trabecular meshwork have similarities in morphology, phagocytosis, expression of extracellular matrix components, and cytoskeleton in vitro [80]. In addition, a three-dimensional model of the trabecular meshwork with perfusion bioreactor technology has been developed to assess glaucoma progression [81]. The primary challenge associated with the utilization of animal models in the study of glaucoma pertains to the multifactorial nature of the disease process and the ability to accurately replicate its symptoms in a patient population [82].

The main goal of gene therapy in glaucoma is to slow the rate of apoptosis of RGCs, but this requires the identification of a suitable neuroprotective agent. One such factor is brain-derived neurotrophic factor (BDNF) [83]. Thus, Osborne et al. demonstrated that the recombinant AAV2-TrkB-2A-mBDNF construct mediates long-term enhancement of neuroprotective BDNF signaling by intravitreal injection, increasing the viability of RGCs in models of optic nerve injury and elevated IOP in vivo. No significant side effects on retinal structure were observed [84]. This approach in the AAV-mediated *TgkB* gene, a receptor for BDNF, may be applicable to optic nerve injury as well [85].

Gene therapy for glaucoma aims to address elevated IOP resulting from impaired aqueous outflow. This condition leads to decreased perfusion, intraocular arterial pressure, and impaired ocular blood flow regulation. Successful studies have been conducted on mice with an ocular hypertension model using an AAV-shh10 construct carrying the CRISPR-Cas9 system and leading to editing of the *AQP1* gene, which plays a crucial role in the production of aqueous humor [86]. In addition, the delivery of the apoptosis regulators *BCL-2* and *BCL-xL* [87] via AAV2, *BIRC4* via AAV [88,89], and *FasL* [90] make it possible to regulate apoptosis and neurodegeneration in glaucoma.

In 2023, O’Callaghan et al. announced the development of a new therapeutic treatment for glaucoma [91]. AAV9-mediated expression of matrix metalloproteinase 3 (MMP3) increased fluid outflow in two glaucoma models in mice and primates. Long-term transduction of corneal endothelial AAV9-MMP3 in primates was found to be safe and well tolerated [91].

A substantial body of evidence is emerging that suggests the potential efficacy of various experimental gene therapies for glaucoma. However, it should be noted that these therapies are still in the early stages of research, and clinical trials on patients using these complex therapies are lacking. An analysis of available PubMed reports (excluding reviews, systematic reviews, and meta-analyses) and the Clinical Trials Database (for interventional studies only) shows a lack of translational studies that could lead to trials of gene therapy in glaucoma [92].

#### 5.1.3. Optic Neuritis

Optic neuritis is one manifestation of central nervous system inflammation with subsequent demyelination. Recurrent episodes of optic neuritis indicate a possible cause for the development of more generalized diseases, including multiple sclerosis, neuromyelitis optical spectrum disorder (NMOSD), and myelin-oligodendrocyte glycoprotein (MOG)-IgG-associated disease [93]. Vision loss associated with optic neuritis in patients with NMOSD and MOG is usually more severe and results in larger scotomas, areas of complete or partial visual field loss. Given the fact that both diseases affect the optic nerve, optic chiasm, and optic tracts, it is not surprising that bilateral visual loss is common. In both cases, the lesion often extends longitudinally into the spinal cord [93].

There are several targets under investigation in gene therapy for optic neuritis-BDNF, which fulfills an important role in the proper formation of retinal structure and neuroprotection. This factor is produced by RGCs, amacrine cells, retinal glial cells, and photoreceptors [94]. In a study in mice with optic nerve injury, intravitreal injection of AAV2-TrkB-2A-mBDNF resulted in an increased viability of RGCs [84].

Neurodegenerative conditions have also been shown to be associated with abnormalities in histone acetylation. In intraocular hypertension, optic nerve damage, ischemia, and hypoxia, histone acetyltransferases are often destroyed, leading to weakening of chromatin structure and regulation of gene expression. However, histone deacetylase activity is greatly enhanced in the above pathological conditions, leading to excess deacetylation activity; gene silencing; and, as a result, physiological dysfunction of nerve cells [95]. Thus, Sun et al. demonstrated a neuroprotective effect after intravitreal injection of liposomes loaded with the histone deacetylase inhibitor, trichostatin A, in a model of optic neuritis in mice. Liposomes reached the medial part of the retina after injection, reducing hyperplasia and apoptosis of RGCs [96].

Regulators of proliferation, viability, cell migration, and genome stability include *PTEN* and *SOCS3*. It has been demonstrated that knockdown of these genes leads to enhanced axon regeneration for 4 weeks after optic nerve injury, promoting proliferation of RGCs [97]. The effect of *PTEN/SOCS3* deletion on the structural integrity of RGC dendrites and axons after optic nerve compression was also demonstrated. It was observed that the deletion of *PTEN* but not *SOCS3* improved the dendritic contraction of RGCs [98].

Another potential target of gene therapy is ciliary neurotrophic factor (CNTF), which induces optic nerve regeneration [99]. Unlike BDNF, a single injection of CNTF protein into the vitreous body can have a significant neuroprotective effect on RGC [100]. In a study by Xie et al., it was demonstrated that AAV2-mediated delivery of CNTF increased CCL5 expression in immune cells and retinal glial cells, and recombinant CCL5 promoted extensive axonal regeneration. However, CRISPR-mediated knockdown of the cognate CCR5 receptor in RGCs or treatment of wild-type mice with a CCR5 antagonist suppressed the effects of CNTF gene therapy [101].

Thus, BDNF, CNTF inhibitors of histone deacetylases, *PTEN* and *SOCS3*, and microRNA-21 may be promising targets in maintaining retinal ganglion cell viability and regeneration [102,103]. To date, 52 clinical trials of gene therapy for optic nerve pathologies have been reported, most of which are aimed at the treatment of Leber’s amaurosis.

### 5.2. Cornea Diseases

**Figure 4:**
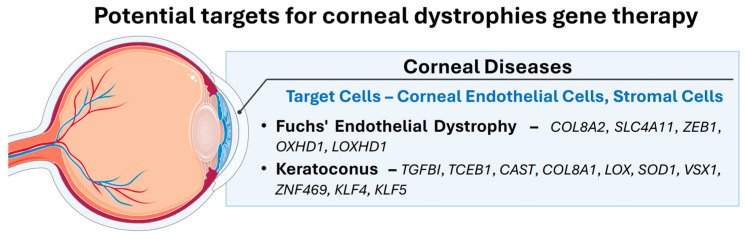
A gene therapy strategy for corneal diseases.

The cornea is susceptible to a variety of injuries and diseases of various etiologies, including genetic mutations, infectious agents, and factors in the development of autoimmune conditions. Gene therapy is used to treat corneal pathologies such as mechanical trauma and chemical burns, infectious keratitis, dry eye syndrome, and corneal dystrophy [104]. Examples of nonhereditary corneal diseases studied for gene therapy include Herpes simplex epithelial keratitis, Sjögren’s syndrome, corneal graft rejection, and corneal neovascularization [4]. Mucopolysaccharidosis, Meesmann corneal dystrophy, Ectrodactyly ectodermal dysplasia-cleft syndrome, aniridia, and corneal endothelial dystrophy (Fuchs’ dystrophy) are among the major inherited diseases that are targets for gene therapy drug development [4]. Fuchs’ corneal endothelial dystrophy and keratoconus are currently the two most frequently studied corneal diseases. The relevance of research on these two pathologic conditions is primarily due to their high prevalence. Moreover, they are the primary indications for keratoplasty in many countries [105]. However, in some patients, corneal transplantation is accompanied by transplant rejection. Moreover, according to data from the WHO, approximately 10–15% of patients remain untreated due to a shortage of donors [106]. The main target genes for gene therapy of corneal diseases are summarized in Figure 4.

#### 5.2.1. Dystrophies of the Cornea of the Eye

Corneal dystrophies are a heterogeneous group of inherited diseases of the cornea accompanied by corneal damage. This pathology is classified depending on the affected corneal layer: epithelial and subepithelial dystrophies, Bowman’s membrane dystrophies, and endothelial dystrophies. Each of these conditions exhibits a unique set of clinical features, variable patterns of inheritance, a distinct age of onset, and varying rates of progression. Corneal dystrophies can be inherited in an autosomal dominant, autosomal recessive, or X-linked manner [106]. Various corneal dystrophies are caused by mutations in the *CHST6* and *KRT* genes, *KRT3* and *KRT12*, *PIP5K3*, *SLC4A11*, *TACSTD2*, *TGFBI*, and *UBIAD1* [107]. Mutations in three genes are known to cause posterior polymorphic corneal dystrophy: *OVOL2*, *ZEB1*, and *GRHL* [108]. Some of the most common diseases of this group are Fuchs’ corneal endothelial dystrophy (FECD) and keratoconus, which will be described in this section.

#### 5.2.2. Fuchs’ Endothelial Corneal Dystrophy

FECD is a bilateral lesion of the corneal endothelium. This disease is characterized by progressive and accelerated corneal endothelial cell death accompanied by several degenerative processes in the Descemet’s membrane [109]. In this pathology, there is an accumulation of aberrant extracellular matrix and the formation of corneal guttae, which is an overgrowth of extracellular matrix on the posterior Descemet’s membrane and causes light scattering, glare, and vision problems [109,110]. Early-onset FECD is associated with mutations in the genes *COL8A2*, *SLC4A11*, *ZEB1*, and *LOXHD1* [106]. Mutations in the *COL8A* gene are associated with an early form of FECD pathology because this mutation affects the structure of the Descemet’s membrane. The *SLC4A11* gene encodes an ion channel that promotes water resorption through the endothelial layer and is an important mediator of solute transport in the cornea [111]. Mutations in this gene can lead to corneal edema and correlate with FECD. Similarly, mutations in the *ZEB1* gene have been associated with late onset of FECD and posterior polymorphous corneal dystrophy [111,112]. Missense mutations in the *LOXHD1* gene are associated with progressive hearing loss and corneal endothelial cell dysfunction in EDRF [111]. Most cases of FECD are caused by expansion of trinucleotide repeats in the *TCF4* gene, resulting in altered mRNA processing due to sequestration of muscleblind-like protein (MBNL)-1 and MBNL2 splicing regulator proteins in nuclear RNA focuses [113].

Currently, the only treatment for FECD is corneal transplantation, which is accompanied by high risk and possible complications. Uehara et al. developed gene therapy based on the CRISPR/Cas9 gene editing system. In a study on mice with an FECD model, they demonstrated that a single intraocular injection of Ad encoding both the *Cas9* gene and guide RNA (Ad-Cas9-Col8a2gRNA) effectively suppressed the expression of mutant *COL8A2* in corneal endothelial cells, prevented endothelial cell loss, and restored the pumping function of corneal endothelium in adult mice [114]. No adverse events were detected by histology and electroretinography.

Corneal endothelial cells exposed to H2O2 can be used as a model in preclinical in vitro tests. Thus, Ceravolo et al. studied the positive effect of polydesoxyribonucleotide in an in vitro model of FECD [115]. Also, a double mutant mouse carrying a tamoxifen-induced Slc4a11 knockdown and Col8a2 mutation was created to establish a comprehensive FECD model for preclinical testing. As a result, increased corneal thickness and decreased endothelial cell density were observed [116].

To date, 79 clinical trials of various therapeutic approaches for Fuchs’ dystrophy have been registered, of which NCT03974230, NCT01795001, and NCT01795001 aim to further explore the molecular pathogenesis of Fuchs’ dystrophy.

#### 5.2.3. Keratoconus

Keratoconus is a bilateral and asymmetric progressive non-inflammatory ectatic corneal disease. The condition is characterized by the presence of cone-shaped thinning of the cornea, which is associated with irregular thinning of the stroma. This results in corneal deformity and degeneration and significant vision loss. The patient’s primary complaints encompass a progressive and variable decrease in visual acuity, accompanied by image distortion and heightened sensitivity to glare and light [117]. The etiology of keratoconus is multifactorial, with environmental and genetic factors playing an important role. A multitude of studies have documented the occurrence of familial aggregation of the disease, thereby suggesting a hereditary component [117]. The most frequently described type of inheritance is autosomal dominant with incomplete penetrance and variable expression [118]. However, the autosomal recessive type of inheritance has also been demonstrated [119]. The whole-genome association studies have also contributed to the identification of several important genetic mutations associated with keratoconus, including the *TGFBI*, *TCEB1*, *CAST*, *COL8A1*, and *LOX* genes [120]. Also, *VSX1*, *SOD1*, and *LOX* genes are associated with keratoconus in some studies [121]. In addition, 36 genetic loci have been identified that are closely associated with keratoconus [122]. Hardcastle et al. identified the essential role of cell differentiation pathways and stem cell regulators (*KLF4* and *KLF5*) in the pathogenesis of keratoconus, as well as the role of genes affecting connective tissue [122].

Among the best-known genes involved in the development of keratoconus is the *VSX1* gene [123], which is also involved in posterior polymorphic corneal dystrophy; the *SOD1* gene [124], involved in defense against free radical-mediated damage; *ZNF469* [125], the mutation of which leads to fragile cornea syndrome [126]; and the signaling pathway of transforming growth factor (TGF)-8 [127], which plays a role in the regulation of extracellular matrix composition [128].

The current treatment method to slow the progression of keratoconus is corneal collagen crosslinking. Nevertheless, this method of prophylactic treatment is not without risk as it can result in endothelial cell damage and associated complications. This is confirmed by the study of Xing et al. in which corneal tissue changes after collagen cross-linking with ultraviolet and riboflavin A were evaluated on New Zealand albino rabbit models [129]. In cases of extreme severity, the implementation of a corneal transplant may be deemed necessary [128]. Another promising treatment is the use of extracellular vesicles derived from mesenchymal stem cells, which have been shown to facilitate tissue regeneration through the delivery of specific factors [130].

To date, no clinical trials of gene therapy for keratoconus have been reported. The multitude of related genomic loci and many comorbidities may be an obstacle to gene therapy, but the identification of the key role of several genes in the pathogenesis of keratoconus may be crucial for the development of a gene therapy drug.

A considerable number of corneal diseases are accompanied by other pathologies. For instance, corneal injury, trauma, or infection can lead to corneal ulceration with concomitant corneal edema, neovascularization, and fibrosis. Consequently, dual gene therapy emerges as a potential treatment modality for addressing the multifaceted clinical manifestations associated with these corneal diseases [131].

### 5.3. Retinal Diseases

**Figure 5:**
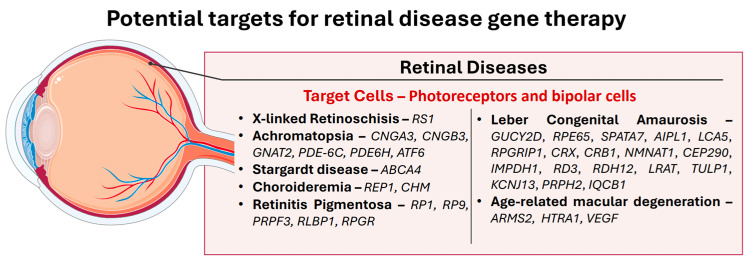
A gene therapy strategy for retinal diseases.

The prevalence and impact of retinal diseases on vision have led to a significant focus on gene therapy research and development in this field. Major retinal diseases include X-linked retinoschisis, achromatopsia, Stargardt disease, choroideremia, RP, LCA, and TMD. This section presents the major retinal diseases, whose targets are summarized in Figure 5.

#### 5.3.1. X-Linked Retinoschisis

X-linked retinoschisis is the most common inherited retinal disease, with an estimated prevalence of 1:5000–20,000 [132]. X-linked retinoschisis is the leading cause of macula lutea degeneration in males, which leads to splitting of the inner retinal layers and visual impairment. Missense mutations, nonsense mutations, frameshift mutations, deletions and insertions, and mutations in splicing sites in the *RS1* gene that cause retinoschisis have now been identified [133]. The *RS1* gene is expressed in bipolar and photoreceptor cells. It is an extracellular lectin that consists of 224 amino acids and binds retinal cell plasma membranes and enables intercellular adhesion as well as signal transduction between photoreceptors and bipolar cells [134,135].

This disease is characterized by symmetrical bilateral lesions of the macula lutea, the debut of which begins in the first decade of life, in some cases, as early as three months of age. Examination of the fundus reveals areas of schisis in the macula, splitting of the layer of retinal nerve fibers, sometimes giving the impression of a pattern of spokes [136].

To date, symptomatic and supportive treatment of X-linked retinoschisis with low-vision aids is provided as visual acuity often deteriorates during the first and second decades of life but remains stable thereafter. In addition, surgical intervention may be required because of the development of complications: intraocular hemorrhage and retinal detachment [136]. Since X-linked retinoschisis is a recessive genetic disease, it may be a potential target for gene replacement therapy. For example, experimental intravitreal gene therapy of AAV resulted in anatomical improvement of the optic nerve and retina on optical coherence tomography and functional improvement of electroretinography (ERG) in X-linked retinoschisis knockout mouse models [137,138].

Veen et al. identified 25 preclinical trials, of which 19 aimed to investigate the efficacy of gene products to deliver intact human or murine RS1 in mouse models of X-linked retinoschisis [139]. To date, five clinical trials of gene therapy for X-linked retinoschisis have been registered [NCT02317887, NCT05878860, and NCT06066008], of which two have been completed [NCT02317354] and have published results [NCT02416622]. In the clinical trial [NCT02416622], intravitreal injection of rAAV2tYF-CB-hRS1 did not result in serious side effects. However, despite good tolerability in patients, no measurable treatment effect was demonstrated.

#### 5.3.2. Achromatopsia

Achromatopsia is a rare genetic disorder inherited in an autosomal recessive pattern with an incidence of approximately 1:30,000 people [140]. This disorder is characterized by decreased visual acuity, pendular nystagmus, increased sensitivity to light, central scotoma, and reduced or complete loss of color vision [141]. This is in contrast to color blindness, in which mutations in genes encoding different photopigments, the type of which divides cones into three groups (S, M, and L types), affect only spectral sensitivity but not the basic function of photoreceptors in the case of achromatopsia [142]. Depending on the form of achromatopsia (complete/incomplete), the function of cones on ERG can be either partially or completely lost; color vision and a higher level of visual acuity are also preserved [143].

In congenital achromatopsia, the retinal phototransduction signaling pathway is impaired, namely, in the inability of cone photoreceptors to respond correctly to a light stimulus by hyperpolarization [144]. Nowadays, six different gene mutations have been identified (*CNGA3*, *CNGB3*, *GNAT2*, *PDE*-*6C*, *PDE6H*, and *ATF6*), of which the most common are *CNGA3* and *CNGB3* [140]. *CNGA3* and *CNGB3* encode the alpha- and beta-subunits of cone cyclic nucleotide-driven ion channels that maintain the membrane potential of cells [145].

Currently, there are no treatments available for achromatopsia, nor any methods to restore full color vision. Physical conservative treatments include the use of vision aids, tinted contact lenses, or glasses to alleviate the symptoms of photophobia [140]. Achromatopsia may be a promising target for gene therapy by transferring a copy of the gene into the affected cells, i.e., photoreceptors and cones.

Based on animal models, it has been suggested that early intervention provides better outcomes compared with older age. Preclinical models of nonhuman primate models with the PDE6C R56Q mutation were tested with AAV5 carrying rhesus PDE6C under the control of the cone-specific promoter PR1.7 [146,147].

To date, seven clinical trials of gene therapy for achromatopsia have been registered: NCT03278873, NCT02610582, NCT02610582, and NCT02599922, in addition to one that has been completed [NCT04124185] and two that have published results [NCT03001310 and NCT03758404]. For example, in a phase I/II clinical trial [NCT03001310], subretinal injection of AAV8-hCARp.h CNGB3 was safe and well tolerated [148]. Favorable changes were observed in individual patients on several assessments, including color vision (26%), photophobia (55%), and improvements in quality of life (91%). Thus, AAV8-hCARp.h CNGB3 gene therapy is a promising approach for the treatment of achromatopsia.

Although AAVs have natural limitations as a gene delivery system, vector engineering can help to develop improved variants of rAAVs that will transduce a larger fraction of photoreceptor cells–cones with higher efficiency and lower immunogenicity and via less invasive routes of administration. Thus, Bertin et al. developed new vector-promoter combinations for efficient transduction of cones in the central fossa because it contains the highest concentration of cones [149].

#### 5.3.3. Stargardt Disease

Stargardt disease is one of the most frequent macular dystrophies with slow progression. The prevalence of this disease is approximately 1:8,000–10,000 [150]. Stargardt disease is inherited in an autosomal recessive pattern and can be caused by mutations in the *ABCA4* gene, which lead to the accumulation of lipofuscin pigment inside retinal pigment epithelium cells, causing degeneration of both retinal pigment epithelium and photoreceptor cells [4]. There are also several other diseases associated with the *ABCA4* gene mutation: cone–rod dystrophy [151], RP [152], and age-related macular degeneration [153].

The *ABCA4* gene encodes rim protein (RmP), a cassette protein that binds ATP to effect changes in the conformation of the ABC transporter. This protein is present on the disc membrane in the outer segment of photoreceptors, rods, and cones and is also involved in vitamin A metabolism [154]. The RmP protein cleaves the N-retinylidene-phosphatidylethanolamine complex formed by the isomerization of 11-cis-retinal into the trans form of retinal as a result of exposure to light photons and by combining with the phospholipid of the disc membranes. In the absence of RmP protein, N-retinylidene-phosphatidylethanolamine complexes accumulate, and N-retinylidene-N-retinylethanolamine is formed because of a biochemical reaction, leading to lipofuscin accumulation in retinal pigment epithelium cells [155]. The high content of N-retinylidene-N-retinylethanolamine slows down the phagocytic ability of retinal pigment epithelium cells, leading them and photoreceptor cells to apoptosis.

New possible approaches to the therapy of Stargardt disease are aimed at reducing the accumulation of lipofuscin. It can be achieved by inhibition of the retinoid cycle—emixustat hydrochloride [156,157]—or by disrupting transporters, retinol binding protein 4 (RBP4), to reduce the formation of toxic bisretinoids. Such drugs include ALK-001 (deuterated vitamin A) [158] and fenretinide (a retinoid-based RBP4 antagonist) [159].

The objective of gene therapy for this disease is to introduce a functional *ABCA4* gene that will promote the expression of active transfer protein in photoreceptor cells, thereby preventing disease progression. However, the AAV widely used in genetic engineering only has a capacity of 4.7 kb, making it insufficient to package the large *ABCA4* gene (7 kb). This problem can be solved by creating a double vector. Thus, the efficiency of tissue culture infection with two AAV vectors carrying the N- and C-terminal fragments of ABCA4 using Cre recombinase was demonstrated [160]. Sun et al. transfected with *PEG-ECO/pGRK1-ABCA4-S/MAR* nanoparticles and demonstrated specific and prolonged expression to *ABCA4* in mouse *Abca4* photoreceptor cells−/−, significantly inhibiting the accumulation of toxic N-retinylidene-N-retinyl ethanolamine in the retina. Inflammation was observed after repeated injections of nanoparticles. This method represents a promising approach of non-viral ABCA4 gene delivery for type I Stargardt disease [161]. In addition, an approach of therapy for this disease based on antisense oligonucleotides to block the produced pseudoexon insertion has been demonstrated [162].

According to the ClinicalTrials.gov database, only one completed clinical trial of gene therapy for Stargardt disease by intravitreal injection of vMCO-010 has been registered [NCT05417126].

#### 5.3.4. Choroideremia

Choroideremia is a rare X-linked inherited chorioretinal dystrophy manifested by progressive degenerative disease of the photoreceptor layer, retinal pigment epithelium, and vasculature [163]. The prevalence is 1 in 50,000 males. Although *REP1* is expressed ubiquitously, only the retinal pigment epithelium layer is affected in patients with choroideremia, leading to the characteristic clinical phenotype of progressive centripetal degeneration [164]. The etiologic factor is a mutation of the *CHM* gene encoding a homolog of the REP1 protein. The symptoms of this disease progress from hemeralopia to loss of peripheral visual fields, with central vision preserved until late in life [165]. Choroideremia is differentiated from X-linked retinitis pigmentosa (RP) as the two diseases may share several features, including nyctalopia, retinal pigment epithelium atrophy, pigmentary changes, reduced ERG, and X-linked inheritance. In contrast to the optic disc pallor seen in PR, in patients with choroideremia, the nerve often has normal anatomy, relative stability of the macula, and peripapillary retina [166].

Gene therapy for choroideremia is constrained by several limitations. Primarily, the paucity of similarity between animal models and the functional and morphological manifestations of the disease constitutes a significant impediment. Additionally, the uncertainty surrounding the most affected retinal layer represents a crucial uncertainty that must be addressed [4].

Antisense oligonucleotides are a promising approach to choroideremia therapy [167]. However, since antisense oligonucleotide therapy depends on the mutation, and a common splicing variant for CHM has not been described, it may not be as widely applicable as other described therapies [168].

The first phase I/II choroideremia gene therapy clinical trial was conducted to test the safety and efficacy of subretinal administration of two doses of the AAV2-REP1 viral vector [NCT01461213]. The initial results demonstrated an improved functioning of the rods and cones, but one of the serious effects of this treatment was retinal detachment. A follow-up phase II clinical trial [NCT02407678] was initiated in 2016, enrolling 30 patients. In 2018, an international phase III clinical trial [NCT03496012] was initiated based on the successful results of the initial phase I/II clinical trial [169]. However, the initial results of the phase III clinical trial of timrepigene emparvovec (BIIB111/AAV2-REP1) did not meet the primary endpoint. The safety results of the study were consistent with previous studies.

A phase I clinical trial of 4D-110 (4D Molecular Therapeutics, Inc., Emeryville, CA, USA) of intravitreal administration of AAV to patients with genetically confirmed choroideremia has also been registered [NCT04483440]. The clinical trial is expected to be completed in 2024.

#### 5.3.5. Retinitis Pigmentosa

RP is a group of diseases accompanied by progressive retinal degeneration, usually beginning in the mid-periphery and spreading to the macula and fossa [170]. RP is the most common inherited retinal dystrophy, affecting more than 1.5 million patients worldwide, and is classified as a rare disease [171,172]. Genetic mutations generally result in the degeneration of rod and cone photoreceptors [173]. Clinical features include hemeralopia, followed by reduced visual fields leading to tunnel vision and total blindness, and an abnormal ocular fundus with bone spicule deposits and weakened retinal vessels [174]. At the cellular level, it correlates with a predominantly affected rod photoreceptor system and subsequent death of cones [170]. The most common form of RP is cone–rod dystrophy, in which the first symptom is hemeralopia, followed by progressive loss of the peripheral visual field in daylight and blindness after a few decades of the patient’s life [175]. With wide clinical and genetic heterogeneity, RP can be inherited as an autosomal dominant, autosomal recessive, or X-linked disease [176].

Achromatopsia, congenital stationary night blindness, and macular dystrophies are some of the retinal dystrophies that have symptoms that overlap with each other in both the eye and the genes. This makes it harder to classify hereditary retinal dystrophies [171]. Also, the heterogeneity among patients with RP is due to the wide range of possible genetic mutations associated with this pathology. Each gene corresponds to a gene-specific subtype of RP with a characteristic age of debut, functional visual impairment, retinal anatomy, and rate of progression. For example, mutations in the genes *RP1*, *RP9*, and *PRPF31* are more characteristic of the autosomal dominant type of inheritance of RP; also, at mutations in the gene *RLBP1*, there are characteristic numerous white dots in the eye fundus [175].

The development of a gene therapy drug for RP requires precise target identification, which is an obstacle due to heterogeneity. In several successful preclinical trials, the most used models have been the mouse model rd9 and the canine model XLPRA2, the latter of which allows the introduction of large numbers of vectors with higher accuracy. The most used vectors have been AAV2/5 and AAV2/8 [177,178,179]; however, CRISPR has recently shown promising results for both model development and pathology reversal [180,181].

Despite this, according to the results of a clinical trial of gene therapy for X-linked RP [NCT03116113] cotoretigene toliparvovec (BIIB112, AAV8-NSR-RPGR), the study did not meet the primary endpoint, but a positive trend was observed for several clinically relevant secondary endpoints. X-linked RP affects primarily male patients and is most associated with mutations in the *RPGR* gene [182]. When the functioning of this gene is disrupted, protein metabolism in photoreceptor cells may be impaired, leading to the disruption of cellular homeostasis and cell death [181]. Importantly, the wild-type *RPGR* gene packaged in AAV vectors contains many spontaneous alterations. Moreover, overexpression of *RPGR* can be toxic and requires careful dosing for gene delivery [183].

An alternative method of gene therapy for RP is the deletion of open reading frame 15 (ORF15) using the CRISPR/Cas9 system with subsequent repair by nonhomologous end joining to restore *RPGR*. Thus, Gumerson et al. confirmed this hypothesis in rd9 mice carrying a frameshift mutation in *RPGR^ORF15^* [181].

To date, 32 clinical trials of gene therapy to find RP have been registered, of which 7 have been completed [NCT04919473, NCT03116113, NCT03252847, NCT01482195, NCT02575430, NCT03780257, and NCT01461213], and 2 trials have been terminated early at the sponsors’ discretion [NCT05176717 and NCT05085964].

One major limitation of gene therapy for RP may be the extent of photoreceptor cell damage. In a study by Scalabrino et al., it was demonstrated that when 20% and 50% of the rods were killed, it was possible to restore normal retinal function, but when the lesions in the retina were more extensive, there was continued, delayed loss of sensitivity and signal transduction in RGCs, as well as persistent gliosis. These findings are an important point in the development of gene therapy for the treatment of RP, as replacement gene therapy provided after a loss of more than 50% of the rods is unable to restore visual function to normal values [184].

#### 5.3.6. Leber’s Congenital Amaurosis

LCA is a group of severe inherited retinal dystrophies with early onset, manifesting progressive visual impairment and blindness. The prevalence of this disease is 1:50,000-30,000 [185]. Several specialists have classified LCA as a severe form of RP [150]. There are several genes associated with *GUCY2D*, *RPE65*, *SPATA7*, *AIPL1*, *LCA5*, *RPGRIP1*, *CRX*, *CRB1*, *NMNAT1*, *CEP290*, *IMPDH1*, *RD3*, *RDH12*, *LRAT*, *TULP1*, *KCNJ13*, *PRPH2*, and *IQCB1* [186]. Most cases are inherited in an autosomal recessive pattern. Due to the wide range of genotypic variability, the clinical phenotypes in carriers of LCA mutations are also highly heterogeneous. The spectrum of ophthalmic disorders encompasses a wide range of presentations, from those that are essentially normal to those characterized by refractive abnormalities, photophobia, nyctalopia, peripheral chorioretinal atrophy, drusen-like deposits, keratoconus, and cataracts. The pathophysiological mechanisms of LCA are associated with impaired phototransduction and the visual cycle and affect the complex homeostatic interaction between photoreceptors and the retinal pigment epithelium layer [187].

To our knowledge, only one gene therapy drug, Luxturna^®^, has been approved to date for *RPE65*-associated LCA, which is associated with mutations in the *RPE65* gene encoding retinoid isomerohydrolase in the retinal pigment epithelium, leading to rod-type retinal dystrophy [188].

Another target for preclinical testing is a mutation in *GUCY2D*, which is associated with severe retinal dystrophy with early onset, LCA1, a major cause of blindness in children. Safety and pre-dependent effects were reported for the transgene with the AAV5 vector (AAV5-hGRK1-GUCY2D). Combined with biodistribution studies in rats and efficacy studies in mice, this provided the basis for the design of the first clinical trial involving humans with LCA1 [189].

In addition, another target is *CEP290*-associated type 10 LCA, whose mutation leads to blindness at an early age. For example, Russel et al. reported a statistically significant improvement in visual acuity and retinal sensitivity by intravitreal injection of sepofarsen, an antisense RNA oligonucleotide. A controlled safety profile was demonstrated in a phase Ib/II clinical trial. The method of gene therapy via LCA is to inhibit the splice site, or mutant sequence, leading to exon skipping or silencing at the mRNA level, the functionality of which is difficult to control through the delivery of gene therapeutic agents by viral vectors. Thus, sepofarsen binds to the mRNA of the *CEP290* gene and returns to normal splicing disrupted by the mutation [190]. To date, an open-label, randomized, controlled, double-blind phase II/III clinical trial of sepofarsen with dose escalation to assess safety and tolerability in children under 8 years of age is underway [NCT04855045].

Another approach in gene therapy for LCA type 10 is to remove the pathogenic splice site using the CRISPR/Cas9 system for normal splicing and expression of the CEP290 protein. A phase I/II clinical trial, EDIT-101, aimed at editing the *CEP290* gene through CRISPR/Cas9 [NCT03872479], is planned to be completed by 2025.

#### 5.3.7. Age-Related Macular Degeneration

Age-related macular degeneration is a chronic inflammatory eye disease affecting the macular region, with a strong hereditary component, usually affecting people over 60 years of age [191]. According to the UN World Population Prospects, by 2040, there will be about 288 million people worldwide with age-related macular lutea degeneration [192]. AMD affects the photoreceptor complex, retinal pigment epithelium, Bruch’s membrane, and the vasculature. This pathology is characterized by the accumulation of optic disc drusen, leading to progressive degeneration of photoreceptors and retinal pigment epithelium, as well as loss of central vision [193]. In addition to drusen, a more diffuse form of deposits, subretinal drusenoid deposits, are commonly seen in the subretinal space [194]. It is important to note that according to Beckman’s classification, the presence of small (less than 63 μm in diameter) solid drusen is considered a sign of biological aging [195]. A distinction is made between early and late stages of AMD, but the age-related eye disease severity scale is often used in clinical studies and trials [196].

Genetic predisposition has been demonstrated to play a significant role in the development of AMD. It is imperative to note that genome-wide association studies have identified a minimum of 103 loci associated with age-related macular degeneration (AMD) [197]. Two genes, *ARMS2* and *HTRA1*, increase the risk of pinuecula degeneration. However, the role of these genes is still not fully understood. The role of *ARMS2* remains ambiguous; initial results suggested a mitochondrial function, and other data suggest that *ARMS2* is a secreted protein and a component of the choroidal extracellular matrix [198]. *HTRA1* encodes a serine heat shock protease and is involved in the regulation of extracellular matrix changes, angiogenesis, the TGF-β signaling pathway, and subretinal inflammation by controlling monocyte elimination [193]. However, the role of these genes in the pathogenesis of AMD is not completely clear, namely, whether mutations in the *ARMS2* gene are associated with the development of late stages of AMD or whether it is caused by coupling with the *HTRA1* gene [199].

Photodynamic therapy, anti-VEGF therapy, is widely used in clinical practice. VEGF promotes neovascularization from the choroid under the retina, leading to retinal edema. Ranibizumab, aflibercept, bevacizumab, and brolucizumab, which are administered by multiple intravitreal injections, are used for the treatment of neovascular AMD [193]. Long-term studies have demonstrated that reductions in signs of AMD achieved in the first two years of therapy do not persist over time for a few reasons [200].

Promising approaches in gene therapy for AMD include RGX-314 (REGENXBIO, Rockville, Maryland), an AAV8 vector expressing a ranibizumab-like anti-VEGF Fab [NCT05407636]; Ix Oberonogene soroparvovec (Ixo-vec) (Adverum Biotechnologies, Redwood City, CA, USA), previously designated ADVM-022, which is an intravitreal (IVT) injection of a proprietary capsid vector, AAV.7 m8, which carries a ubiquitination cassette encoding the aflibercept protein [NCT05536973]; and 4D-150 (4D Molecular Therapeutics (4DMT), Emeryville, CA, USA), which is a transgenic cassette expressing aflibercept and a VEGF-C inhibitory miRNA that effectively suppresses VEGF A, B, C, and placental growth factor [NCT05197270] [201].

To date, 37 AMD gene therapy clinical trials have been registered, of which 8 have been completed [NCT05417126, NCT03748784, NCT01494805, NCT03066258, NCT01024998, NCT03585556, NCT03144999, and NCT04418427].

## 6. Conclusions and Prospects

Gene therapy for ocular diseases continues to develop, and new technologies and advances may determine the future direction of the field. In addition to the abovementioned approaches, such technologies also include optogenetics, which is a promising method for the treatment of degenerative retinal diseases, such as RP. Optogenetics is based on the introduction of light-sensitive proteins–opsins into retinal cells to restore vision in the case of malfunctioning or death of photoreceptor cells [202,203].

Importantly, gene therapy requires the delivery of a targeted gene or system like CRISPR/Cas9 via vectors to alter the functioning of the genetic machinery of the target cell. Despite the several advantages of this method, safety, route of administration, toxicity, efficacy, and dosage of viral particles are of primary importance before FDA approval and drug registration. With continued research into the use of various vectors in gene therapy, new generations of modified viruses and non-viral vectors are emerging as potential carriers with greater transduction efficiency, tropism to target cells, and fewer side effects. Personalized gene–cell therapy with the introduction of genetically modified autologous cells of the patient may serve as a possible solution to the development of toxicity.

Another important aspect for the development of a gene therapy drug is the selection of a target gene, especially for the therapy of optic nerve damage, glaucoma (depending on its type), FECD, keratoconus, achromatopsia, and LCA.

Additionally, the representation of positive and successful in vitro results in larger animal models and, most notably, in humans is of great importance. This is due to the presence of anatomical and biochemical differences. Further research is necessary to advance the field of gene therapy for ocular diseases. Specifically, the creation of models of various pathological conditions represents a significant challenge and a major limitation. This problem is being addressed by tissue engineering with the development of drug test systems on 2D models using immortalized cell lines, 3D models such as spheroids, organoids, organs on chips, and in silico analysis [204]. Another significant limitation is the important issue of the duration of gene therapy in the eye. For example, according to a systematic review and meta-analysis by Sobh et al., long-term visual improvement in LCA and LHON from 3 to 11 years was demonstrated, but progression of degeneration of the affected areas was also observed [205]. Thus, further long-term studies are needed to monitor functional improvements because of gene therapy.

In addition, the high cost of gene therapy set by pharmaceutical companies has raised concerns among patients and doctors. For example, at the time of its release, the price of Luxturna was USD 425,000 for an injection in one eye [206]. Certainly, clinical development of a genotherapeutic drug requires time and money, which can be justified by potential clinical efficacy. One of the main risks for pharmaceutical companies is the rare occurrence of a particular ophthalmologic disease. For example, despite approval in Europe, the clinical use of Glybera^®^ in the treatment of lipoprotein lipase deficiency was discontinued due to the lack of demand for a rare monogenic inherited disease [207]. In the context of developing a genotherapeutic drug for ophthalmologic diseases, it is imperative to identify a precise target, as is the case with glaucoma or keratoconus.

In this review, the prospects of gene therapy for ophthalmic diseases were considered. It is only through a comprehensive understanding of the anatomical, molecular, and genetic underpinnings of pathologies that we can effectively expand the potential applications of gene therapy to restore vision in patients.

## Figures and Tables

**Table 1:** List of approved and ongoing clinical trials of AAV-based gene therapy for various eye diseases.

Disease.	Viral Vector	Type of Injection	Phase	Identifier Number	Study Status
Leber congenital amaurosis	AAV2-RPE65	Subretinal	I	NCT00516477	Completed
			I/II	NCT01208389	Active, not recruiting
			III	NCT00999609	Active, not recruiting. With results
			Observational	NCT03602820	Active, not recruiting
			Observational [patient registry]	NCT03597399	Active, not recruiting
	ASO	Intravitreal	I/II	NCT03140969	Completed
			I/II	NCT03913130	Terminated [study prematurely terminated due to sponsor decision for reasons unrelated to safety]
			II/III	NCT03913143	Active, not recruiting
			II/III	NCT04855045	Recruiting
X-linked retinoschisis	AAV2-hRS1	Intravitreal	I/II	NCT02416622	Completed
	AAV8-scRS		I/IIa	NCT02317887	Active, not recruiting
Achromatopsia	AAV2-CNGA3	Subretinal	I/II	NCT02935517	Active, not recruiting
	AAV2/8-CNGB3		I/II	NCT03278873	Active, not recruiting
			I/II	NCT03758404	Completed
			I/II	NCT03001310	Completed
Wet macular degeneration	AAV2-aflibercept	Intravitreal	I	NCT03748784	Completed
			Observational	NCT04645212	Active, not recruiting
			II	NCT05536973	Active, not recruiting
	AAV2-VEGF		I/II	NCT05197270	Recruiting
	AAV2-sCD59		I	NCT03585556	Completed
Diabetic macular edema	AAV.7m8-aflibercept	Intravitreal	Observational	NCT05607810	Enrolling by invitation
	AAV.7m8-aflibercept		II	NCT04418427	Completed
	AAV2-RPGR		I/II	NCT04517149	Active, not recruiting
Cone–rod dystrophy	AAV-RdCVF/L	Subretinal	I/II	NCT05748873	Recruiting
X-linked retinitis pigmentosa	AAV2/5-RPGR	Subretinal	I/II	NCT03252847	Completed
	AAV8-RPGR		III	NCT03584165	Enrolling by invitation
	AAV2-RPGR	Intravitreal	I/II	NCT04517149	Active, not recruiting
Choroideremia	AAV2-REP1	Subretinal	III	NCT03584165	Enrolling by invitation
	AAV2-REP1		II	NCT03507686	Completed
			III	NCT03496012	Completed
	AAV2-CHM	Intravitreal	I	NCT04483440	Active, not recruiting
Neovascular age-related macular degeneration	AAV8-anti-VEGF Fab	Intravitreal	I/II	NCT03066258	Completed
Retinitis pigmentosa	ASO	Intravitreal	II	NCT05085964	Terminated (business decision)
	ASO		I/II	NCT03780257	Completed
			II/III	NCT05176717	Terminated (business decision)
	AAV2-ChR2		I/II	NCT02556736	Active, not recruiting
	AAV8-RLBP1	Subretinal	I/II	NCT03374657	Active, not recruiting
Geographic atrophy secondary to age-related macular degeneration	AAV2-CFI	Intravitreal	II	NCT05481827	Enrolling by invitation
Leber hereditary optic neuropathy	AAV2/2-ND4	Intravitreal	I/II	NCT02064569	Completed
			III	NCT03406104	Completed

RPE65—retinal pigment epithelium-specific 65 kDa protein; ASO—antisense oligonucleotide; RS—retinoschisin; CNG—cyclic nucleotide gated channel; VEGF—vascular endothelial growth factor; sCD59—soluble CD59; RPGR—retinitis pigmentosa GTPase regulator; RdCVF/L—rod-derived cone viability factor, long thioredoxin-like isoform; REP1—Rab escort protein 1; ChR2—channelrhodopsin-2; RLBP1—retinaldehyde-binding protein 1; CFI—complement factor I; ND4—NADH-ubiquinone oxidoreductase chain 4 protein.
